# A Novel Molecular PCM Wall with Inorganic Composite: Dynamic Thermal Analysis and Optimization in Charge–Discharge Cycles

**DOI:** 10.3390/ma16175955

**Published:** 2023-08-30

**Authors:** Qianru Yang, Jianwu Xiong, Gang Mao, Yin Zhang

**Affiliations:** School of Architecture, Southwest Minzu University, Chengdu 610225, China

**Keywords:** PCM, building wall, dynamic heat transfer, melting fraction, charge–discharge cycle

## Abstract

The combination of electric heating and thermal energy storage (TES) with phase change material (PCM) can achieve load shifting for air conditioning energy saving in building sectors. Their non-flammability, relatively good mechanical properties, and low cost make inorganic PCMs attractive in construction engineering. However, PCMs often show poor thermal conductivity, low heat transfer efficiency, leakage risk, etc., in applications. Moreover, the practical thermal performance of PCM–TES sometimes fails to meet demand variations during charge and discharge cycles. Therefore, in this study, a novel integrated electric PCM wall panel module is proposed with quick dynamic thermal response in space heating suitable for both retrofitting of existing buildings and new construction. Sodium–urea PCM composites are chosen as PCM wall components for energy storage. Based on the enthalpy–porosity method, a mathematical heat transfer model is established, and numerical simulation studies on the charge–discharge characteristics of the module are conducted using ANSYS software. Preliminary results show that the melting temperature decreases from 50 °C to approximately 30 °C with a 30% urea mixing ratio, approaching the desired indoor thermal comfort zone for space heating. With declining PCM layer thickness, the melting time drops, and released heat capacity rises during the charge process. For a 20 mm thick PCM layer, 150 W/m^2^ can maintain the average surface temperature within a comfort range for 12.1 h, about half the time of a 24 h charge–discharge cycling periodicity. Furthermore, placing the heating film in the unit center is preferable for improving overall heat efficiency and shortening the time to reach the thermal comfort temperature range. This work can provide guidance for practical thermal design optimization of building envelopes integrated with PCM for thermal insulation and energy storage.

## 1. Introduction

### 1.1. Background

With economic growth and improved living standards, the building industry consumes 50% of the world’s electricity and accounts for 38% of total global carbon emissions [1]. In order to ensure building occupants’ thermal comfort, maintaining the indoor thermal environment with clean, energy-efficient and low-emissions air conditioning systems is important [2]. An electric radiant heating system is a low-temperature heating system that saves space, does not generate noise, and can provide even temperature distribution, which is beneficial for improving thermal comfort [3]. For instance, radiant floors significantly extend the thermal response time and counter changes in envelope and room load thermal parameters. Such systems offer excellent space utilization, uniform temperature distribution, thermal comfort, and low energy consumption. With the implementation of peak and valley electricity prices, it is imperative to achieve the transfer of daytime electric heating loads to nighttime [4]. The increasing challenges of clean and reliable building energy consumption, along with the pressure of accelerating urbanization and city expansion, highlight the need for green transformation of city building energy supply and consumption chains, especially in developing countries like China [5]. Thus, in the context of the “double carbon” strategy in China initiated in 2020 to achieve net-zero CO_2_ emissions by 2060, Chinese cities have been striving to optimize building energy systems in terms of changes from a combustion-fuel-based structure to a safe and reliable electricity-based system, integrating renewable energy exploitation and energy storage technologies [6].

However, in practical engineering fields, a considerable gap is often observed between building energy supply and terminal load demands, both in terms of time and the amount of energy. Such a dynamic mismatch problem inevitably contributes to low energy utilization efficiency. Integrating thermal energy storage (TES) technology with building energy systems can effectively achieve peak load shifting, which is not only favorable for achieving economic benefits based on peak–valley electricity prices for users but also beneficial for stable and reliable operation of city power grids [7]. TES has become a convenient off-the-shelf approach for building energy saving. For instance, one of the most attractive and effective technologies for heat storage in buildings is the use of phase change materials (PCM), either in building envelope application or for latent heat storage in various air conditioning systems [8]. Such PCM–TES systems have relatively high energy storage density and maintain a nearly constant temperature during phase change, thereby improving thermal comfort [9]. Coupled with new materials and improved systems, integrated PCM building applications have become increasingly attractive from both research and engineering perspectives [10].

### 1.2. Literature Review

**Figure 1:**
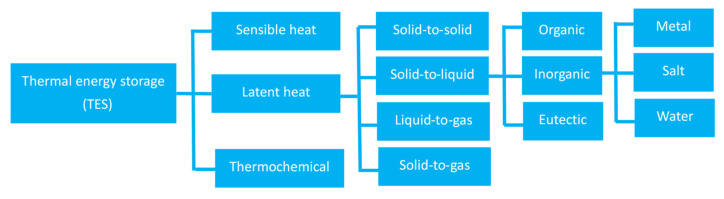
TES technology classification with a focus on materials [11,12,13].

Thermal energy storage (TES) transfers heat to storage media during the charge period and releases it later during the discharge stage. TES has been widely utilized in renewable energy projects, industrial processes, building services, and environmental systems in combination with a variety of energy storage temperatures, device configurations, materials, media, etc. [11]. Combining TES equipment with energy supply and conversion systems has proven to be an effective measure to increase thermal or economic efficiency via the load-shifting function [12]. As shown in Figure 1, sensible heat, latent heat and thermochemical storage take three typical TES forms relying on temperature, phase transition, and chemical energy, respectively [13]. Compared to sensible TES, latent TES with phase change materials (PCMs) has relatively high energy storage capacity and density, making it increasingly attractive for high-energy-density applications [13].

With rapid industrialization and technological development, the applications of various PCMs have drawn increasing attention in recent years, especially in the architecture and construction sectors [14]. Kitagawa et al. [15] pointed out that among the available phase transition materials or composites, solid–liquid PCMs, such as paraffin, salt hydrates, fatty acids, and ester’s are applicable in building applications due to their relatively minimal volume variation during the phase change process, along with their desirable melting and freezing features. Gonzalez et al. [16] and Zhang et al. [17] further stated that phase change temperature and latent transition heat are the two main considerations in engineering fields, considerably impacting the energy storage capacity and thermal application performance during charge and discharge processes. Some organic and inorganic PCMs considered in the existing literature are listed in Table 1 [13,14,17].

Li et al. [18] and Cheng et al. [19] chose paraffin as a PCM for energy storage in building heating, cooling, and ventilation systems. They found that paraffin has a desirable melting temperature approaching the indoor thermal comfort zone according to building thermal design codes and standards (e.g., 18–26 °C based on ASHRAE Standard 169-2013 [20]. Zeng et al. [14] obtained the optimal phase change temperatures and enthalpies under different climatic conditions using an inverse problem algorithm. However, the authors also found that the heat release capacity of paraffin is relatively low during the solidification process, either stabilized or encapsulated. On the other hand, the phase separation supercooling problem contributes to a low heat transfer rate for energy discharge, making it difficult to meet timely load demand variations [21]. The mechanical properties and flammability of most organic PCMs such as paraffin also impose significant restrictions in building applications for safety reasons [22].

In recent years, many researchers have investigated the synthesis of new PCMs, systems and devices, as well as the improvement of thermophysical properties of existing materials [23,24]. Prabakaran et al. [25] studied the thermodynamic features and environmental impacts of an air conditioning system with HFO-1234yf phase change and concluded that it is a suitable alternative to traditional refrigerant in terms of both its coefficient of performance and exergy efficiency. Kim et al. [26] measured the stability, density, rheology, and thermal conductivity of carbon-nanotube-based PCM nanocomposites through experimentation. The authors applied the PCMs under study to building indoor air conditioning with a phase change temperature of 8–10 degrees and achieved thermal conductivity augmentation in both liquid and solid states because of the heat transfer impact from the carbon-assisted phase change nanocomposite. Subsequently, Kumar et al. [27] synthesized nanocomposites made of phase change material (PCM) and multiwall carbon nanotubes in varying concentrations. The experimental results indicated that the droplet contact angles of the nanocomposites increased with increasing volume concentration.

Compared to ordinary materials with constant thermophysical properties, PCMs often show high non-linear and non-uniform heat transfer features due to their density, specific heat, and thermal conductivity changes during phase transition processes [14,17,28]. Therefore, accurate heat transfer modeling is important for thermal performance simulation of PCMs, with the consideration of dynamic parameter changes in charge–discharge cycles. Zhou et al. [29] presented a state-of-the-art review of novel PCM-based strategies for building cooling performance enhancements and thermal modeling methods for energy performance pre-estimation. Munoz et al. [30] studied earth-based materials and straw bales for architectural design and built an evaluation model to assess energy consumption, thermal comfort, mechanical response, and environmental impact. A typical detached house was modelled according to Chilean building codes, and an energy assessment was conducted via dynamic calculations, with environmental impact determined using the ReCiPe methodology. Jin et al. [31] and Sun et al. [32] investigated the heat transfer mechanisms of both distributed and coupled PCM systems to provide an in-depth understanding and presented solutions for system performance enhancement of novel PCM-based systems. The authors also presented a systematic overview of novel PCM-based strategies for thermal performance enhancement, together with the technical challenges of widespread applications.

### 1.3. Objective and Focus

Although existing studies have reported a variety of materials, technologies, systems, and methods for PCM–TES usage in the building sector, practical PCM application effects still differ from pre-estimated effects, considering the complexity of different load demands. For instance, as thermal energy storage media, quick dynamic thermal responses to charge or discharge requirements are preferable. In particular, for solid-to-liquid PCMs, the phase transition characteristics show some disparities between melting and solidification processes, as the thermophysical properties continue to change during charge–discharge cycles. As a result, meeting stored and released heat energy capacity demand is not an easy task, both in terms of time and the amount of energy required. Moreover, the non-linear and non-uniform heat transfer features of integrated PCM building envelopes make accurate simulation and estimation of dynamic thermal performance difficult, especially considering the thermophysical differences among PCM composites.

Therefore, we pose the following questions: How can potential PCM composites be made more feasible for building TES applications? What is the heat transfer impact mechanism behind integrated PCM wall units? Given the dynamic thermal responses in charge–discharge cycles, how can PCM–TES thermal design be optimized? In order to tentatively address these academic problems, in this paper, we consider an inorganic sodium acetate–urea composite as a basic component for energy storage and propose a novel integrated electric PCM wall panel module. Three steps are followed: (1) The key thermophysical properties of PCM composites are tested under different mixing ratios, with emphasis on the melting temperature and super-cooling degree variations during the solidification process. (2) Based on the enthalpy–porosity method, a mathematical heat transfer model is established for thermal performance simulation, and numerical dynamic studies on charge–discharge characteristics of the module are conducted using ANSYS software (Ansys–Fluent v22.2). (3) Key design parameters of the proposed PCM wall are comparatively analyzed and investigated, including the thickness, location, and heating capacity of the PCM layer, considering dynamic melting fraction variations and non-uniformity during the phase transition process. This work can provide theoretical and methodological support for thermal performance estimation of PCM–TES systems, offering guidance for the design of PCM optimization for building applications.

## 2. Methods

### 2.1. Molecular PCM Wall unit with Sodium Acetate–Urea Composite

**Figure 2:**
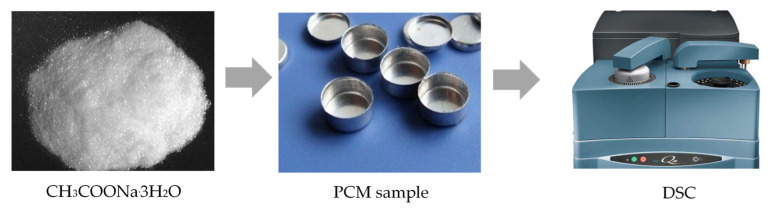
DSC testing for thermal properties of sodium acetate as a PCM.

**Figure 3:**
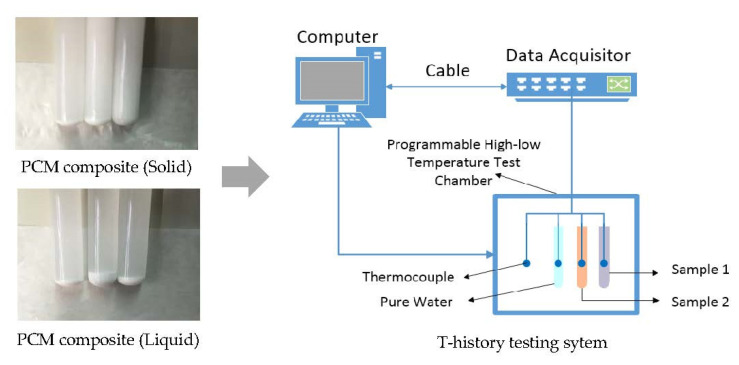
T-history method for thermal property testing of sodium acetate–urea composite [17].

In this paper, sodium acetate is chosen as a basic inorganic PCM, with a phase change temperature ranging from 45 to 55 °C. According to previous studies, its melting temperature approaches the indoor thermal comfort zone (i.e., 18–26 °C), making sodium acetate thermally desirable in PCM–TES applications to achieve energy savings in buildings [17]. Thus, sodium acetate–urea composite was synthesized in the present study, making use of the temperature adjustment effect of mixing urea, with minimal changes in latent transition heat. Figure 2 shows the pure sodium acetate sampling and testing process using a differential scanning calorimetry (DSC) device. According to available research, the main shortcoming of the DSC method lies in the sample volume restriction (e.g., less than 10 mg) [19]. Nonetheless, for most salt hydrates, the small sample amount often makes the subcooling problem more obvious during the solidification process, especially for inorganic composites [28]. Figure 3 shows a schematic diagram of the T-history approach for thermophysical property testing of the synthesized sodium acetate–urea composites with different mixing ratios.

**Figure 4:**
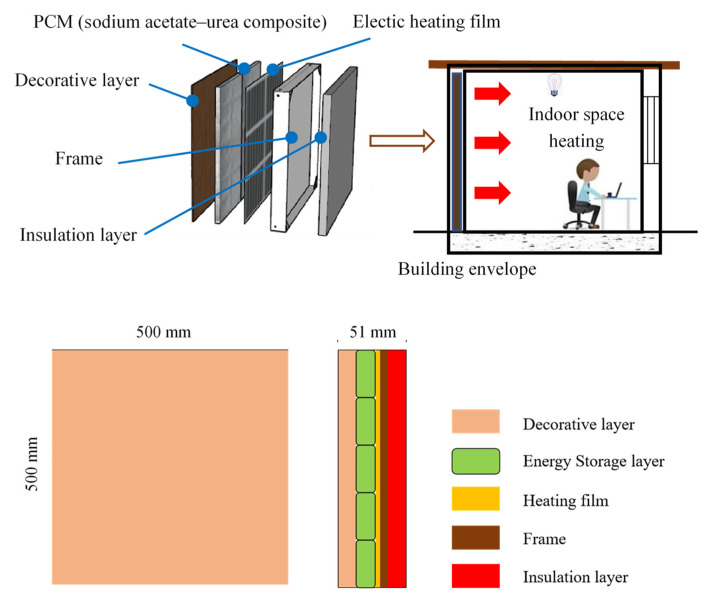
Structure of the proposed modular PCM wall unit.

In practical engineering applications, such inorganic PCM composite particles are often encapsulated into supporting containers called shape-stabilized phase change material in order to avoid leakage. Such supporting containers can remain in a fixed state, even if the working materials change from solid to liquid, especially when used in building envelopes [17,28]. Figure 4 shows the prototype of the proposed molecular PCM wall unit, which is composed of multiple layers (decorative, electric film, PCM, frame, and insulation) and can be inserted into the external walls of buildings for indoor thermal environmental control and space air conditioning with load-shifting considerations. The dimensions of the modular phase change material wall panel are 500 mm × 500 mm, with elements including (from left to right) the finish layer, thermal storage layer, electric thermal membrane, frame, and insulation layer. The physical parameters of each layer are shown in Table 2.

### 2.2. Dynamic Heat Transfer Modeling

To simplify numerical analysis, the following assumptions were made [33]:

- The motion of melted PCM is considered a Newtonian incompressible laminar flow;
- Thermophysical properties of the PCM are independent of temperature;
- Boussinesq approximation is invoked to model buoyancy-induced natural convection;
- All materials are regarded as homogeneous and isotropic in all directions;
- PCM volume change during the phase transition is negligible;
- Contact surfaces are closely fitted, the contact thermal resistance of the interface is zero, and the temperature and heat flow are continuous;
- The thickness of the electric heating film is zero.

The enthalpy–porosity method was used to numerically simulate the phase change heat transfer process within the rectangular cavity using the following governing equation for the 2D model [17,33]. The mass continuity equation and the momentum equation in the *x* axis and *y* axis directions, respectively, are expressed as follows:

(1) $$\frac{\partial u}{\partial x}+\frac{\partial v}{\partial y}=0$$

(2) $$\frac{\partial (\rho u)}{\partial t}+\frac{\partial (\rho uu)}{\partial x}+\frac{\partial (\rho uv)}{\partial y}=-\frac{\partial P}{\partial x}+\left[\frac{\partial }{\partial x}\left(\mu \frac{\partial u}{\partial x}\right)+\frac{\partial }{\partial y}\left(\mu \frac{\partial u}{\partial y}\right)\right]+S_{x}$$

(3) $$\frac{\partial (\rho v)}{\partial t}+\frac{\partial (\rho vu)}{\partial x}+\frac{\partial (\rho vv)}{\partial y}=-\frac{\partial P}{\partial x}+\left[\frac{\partial }{\partial x}\left(\mu \frac{\partial v}{\partial x}\right)+\frac{\partial }{\partial y}\left(\mu \frac{\partial v}{\partial y}\right)\right]+\rho \beta \left(T-T_{m}\right)+S_{y}$$

where *u* and *v* are the velocity (m/s) in the *x* and *y* directions, respectively; *ρ* is the density of the PCM (kg/m^3^); *μ* is the dynamic viscosity of the PCM (Pa·s); *β* is the coefficient of thermal expansion of the PCM (1/K); *T_m_* is the average value of the temperature of the phase change material (K); and the acceleration due to gravity (*g*) is assumed to be −9.8 m/s^2^. In Equations (1) and (2), *S* is the source term related to the liquid fraction of PCM in the pore volume, and *S_x_* and *S_y_* are the components of the source term in the *x* and *y* directions, respectively, which are defined as follows:

(4) $$S_{x}=-A_{mush}\frac{{\left(1-\gamma \right)}^{2}}{\gamma^{3}+\sigma }u$$

(5) $$S_{y}=-A_{mush}\frac{{\left(1-\gamma \right)}^{2}}{\gamma^{3}+\sigma }v$$

where *A_mush_* is the mushy zone constant related to the morphology of the mushy region, the value of which is in the range of 10^4^~10^7^ kg/(m^3^·s). As discussed in Section 2.1, *A_mush_* is 10^5^ kg/(m^3^·s); *σ* is a small value to avoid errors arising from division by zero and is set as 10^−3^; and *γ* is a liquid fraction, which can be calculated according to the following equation:

(6) $$\gamma =\left\{\begin{array}{c} 0\;,\;T\leq T_{s}\;(\mathrm{solid}\;\mathrm{phase})\\ (T-T_{s})/(T_{l}-T_{s})\;,T_{s}<T<T_{l}\;\\ 1\;,T\geq T_{l}\;(\mathrm{liquid}\;\mathrm{phase}) \end{array}\right.(mushyzone)$$

where *T_s_* and *T_l_* are the solidus and liquid temperature (K), respectively. The enthalpy method is used to derive the energy balance equation of the PCM layer:

(7) $$\frac{\partial (\rho H)}{\partial t}+\frac{\partial (\rho uH)}{\partial x}+\frac{\partial (\rho vH)}{\partial y}=\frac{\partial }{\partial x}\left(k\frac{\partial T}{\partial x}\right)+\frac{\partial }{\partial y}\left(k\frac{\partial T}{\partial y}\right)$$

(8) $$H=h_{ref}+\int_{T_{ref}}^{T}c_{p}dT+\gamma h_{sf}$$

where *c_p_* is the specific heat capacity (J/kg·K), *k* is the thermal conductivity (W/m·K), *h_ref_* is the reference enthalpy (J/kg), *T_ref_* is the reference temperature (K), *h_sf_* is the latent heat (J/kg), and ∆*H* equals 0 when the PCM is solid and *h_sf_* when the PCM is liquid. The governing equation for the other wall layers is as follows:

(9) $${\left(\rho c_{p}\right)}_{i}\frac{\partial T}{\partial t}=k_{i}(\frac{\partial^{2}T}{\partial x^{2}}+\frac{\partial^{2}T}{\partial y^{2}})$$

The initial conditions and boundary conditions of the model are expressed as follows:

(10) $${\left.\frac{\partial T}{\partial t}\right|}_{t=0}=T_{init},{\left.-k\frac{\partial t}{\partial x}\right|}_{x=0}=h_{d}\left(T-T_{in}\right),\;{\left.-k\frac{\partial t}{\partial y}\right|}_{y=0}=0,\;{\left.-k\frac{\partial t}{\partial x}\right|}_{x=0.028}=q_{r}$$

## 3. Results and Discussion

### 3.1. PCM Thermophysical Property Testing

**Figure 5:**
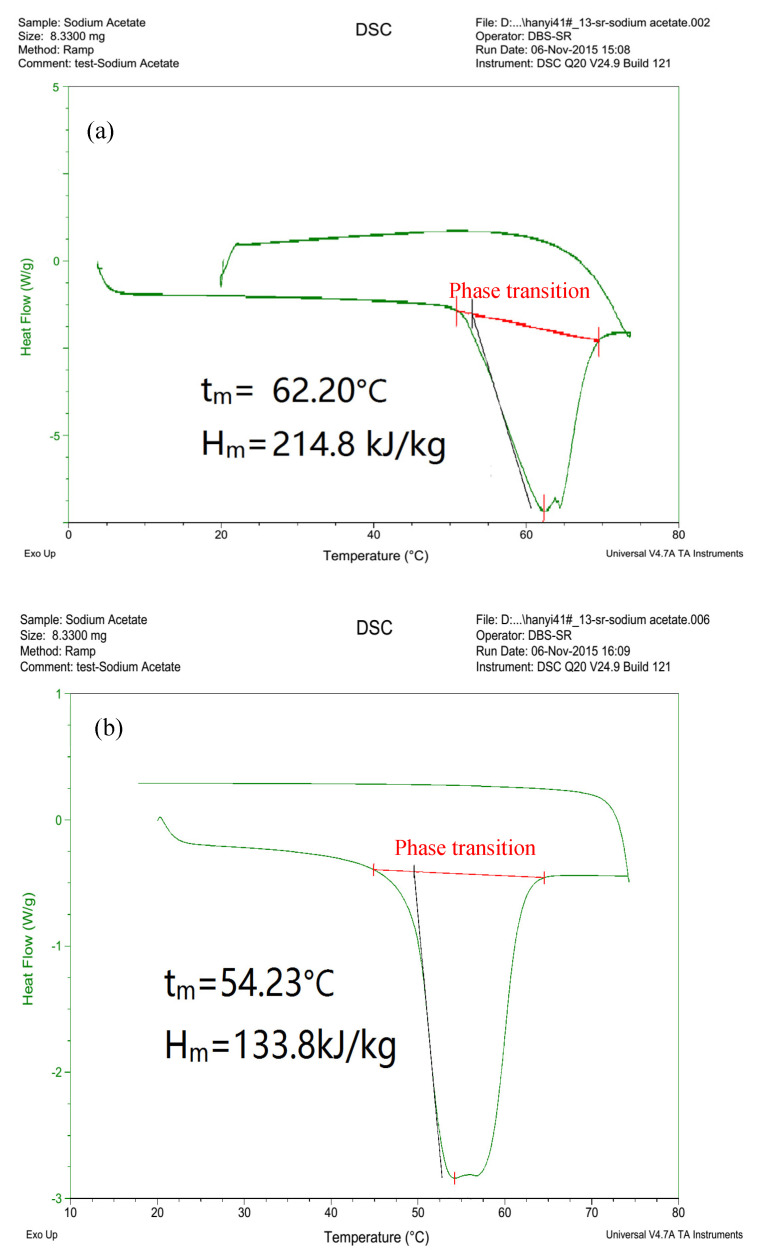
DSC test results of sodium acetate in (**a**) the first cycle and (**b**) the fifth cycle.

**Figure 6:**
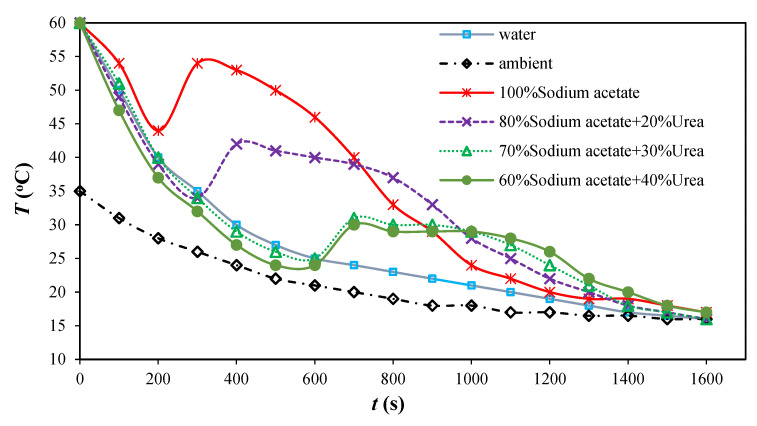
Temperature variation curves for sodium acetate–urea PCM determined using the T-history method.

**Figure 7:**
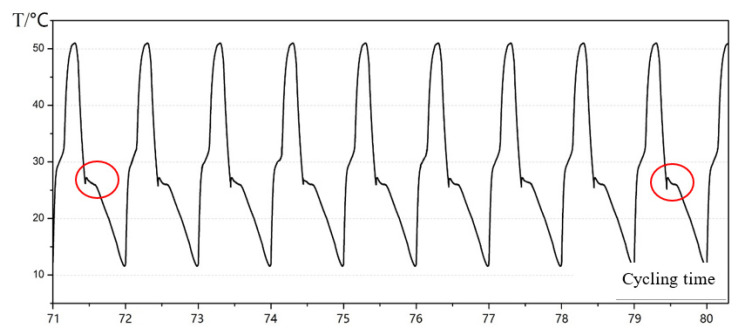
Repetitive temperature tests in PCM composite charge–discharge cycles (red circle: thermal stability comparison during phase transition).

Figure 5 shows the tested thermophysical properties of a pure sodium acetate sample (8 mg) determined via a DSC device. The phase change temperature ranges from 54–62 °C, which is slightly higher than the indoor thermal comfort zone for space heating. However, the measured value is inconsistent with both the melting temperature and phase change latent heat shrinkage after several charge–discharge cycles. Hence, sodium acetate–urea PCM composites were synthesized to adjust the melting temperature and enhance thermal stability. Figure 6 illustrates the temperature variation curves of PCM composites with respect to water and ambient air temperature changes as references. With increased urea fraction, both the phase change temperature and latent heat (enthalpy) decrease slightly. Compared to pure sodium acetate, the melting temperature declines from 50 °C to 28–30 °C with a 30% urea mixing ratio, approaching the indoor air temperature and making it suitable for building-associated thermal energy storage. Furthermore, such a PCM composite can mitigate the supercooling problem during the solidification process, which is beneficial in terms of facilitating heat release during the discharge process in TES applications. As shown in Figure 7, the urea content also contributes to the improvement in thermal reliability and stability in charge–discharge cycles, with only approximately 4% thermal performance decay ratio after 80 cycles with the PCM composite.

### 3.2. Numerical Case Analysis

**Figure 8:**
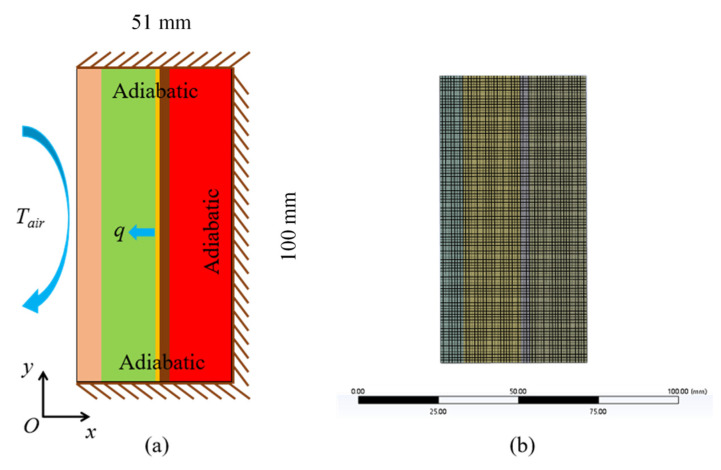
Numerical simulation: (**a**) heat transfer model and (**b**) mesh grid.

In order to investigate and predict the performance of the proposed PCM wall under different structures and operating conditions, Ansys-Fluent v22.2 commercial software was used for numerical calculations. The simplified heat transfer model is shown in Figure 8. One-fifth of the original model was excluded for simulation, and a structured mesh was used to discretize the physical model. The mesh was imported into Fluent for numerical solution. The second-order windward format was chosen to discretize the energy and momentum equations. The laminar flow model was chosen. In the solidification/melting model, *A_mush_* was set to 1 × 10^5^, the transient term was discretized in second-order fully implicit format, the momentum and energy terms were discretized in second-order windward differential format, the pressure term was discretized using the PRESTO algorithm, and the coupling of the pressure and velocity was discretized using the SIMPLE algorithm. The pressure relaxation factor was set to 0.3, the momentum equation relaxation factor was set to 0.7, and the energy equation relaxation factor was set to 1. The residuals were set to 10^−3^, 10^−3^, and 10^−6^ for the continuity equation, momentum equation, and energy equation, respectively.

**Figure 9:**
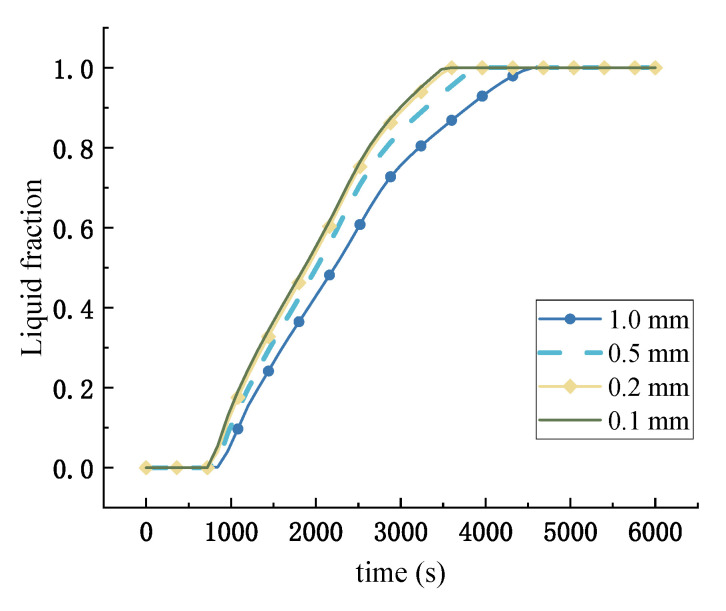
Independent test of grid number for computational fluid dynamics.

The grid step (0.1 mm, 0.2 mm, 0.5 mm, and 1 mm) and time step (5 s, 15 s, 30 s, and 60 s) were analyzed for independence, as shown in Figure 9. Grid sizes of 510,000, 127,500, 20,400, and 5100 cells were considered. The average error with a grid step of 0.2 mm is less than 5% compared with a grid step of 0.1 mm, so a grid step of 0.2 mm (127,500 cells) was considered in all cases in this study. Similarly, the time step was set to 30 s.

As shown in Table 3, in order to study the influence mechanism of heating power, the thickness of the PCM, the layer position, and the heat charge and discharge time of the proposed PCM wall, four sets of simulated conditions (A, B, C, and D) were set up, with three comparison cases for analysis of each factor. *d* is the thickness of the PCM, *q* is the heating power, R represents a setup in which the electric heating film is on the right side of the PCM, L represents a setup in which the electric heating film is on the left side of the PCM, and M represents the middle PCM position. *t*_charge_ represents the heating time, whereas *t*_discharge_ represents the duration of heating stoppage. According to the ASHRAE standard (ASHRAE 55-2020 [34]), when using low-temperature radiant floor heating, the average temperature of the ground under areas of frequent human activity should be 25~27 °C and not exceed 29 °C, whereas that of the ground under areas of occasional human activity should not exceed 32 °C [7]. The efficiency of a PCM wall in 24 h can be calculated using Equation (11), where *q* is the heat flow of the heat transfer to the room, *q_r_* is the surface heating flow, and all input power of the heating film is converted into heat.

(11) $$\varphi =\frac{\int_{0}^{86,400}qdt}{q_{r}t}=\frac{\sum_{0}^{2880}{30q}_{i}}{86,400q_{r}}$$

### 3.3. Dynamic Phase Change Simulation

**Figure 10:**
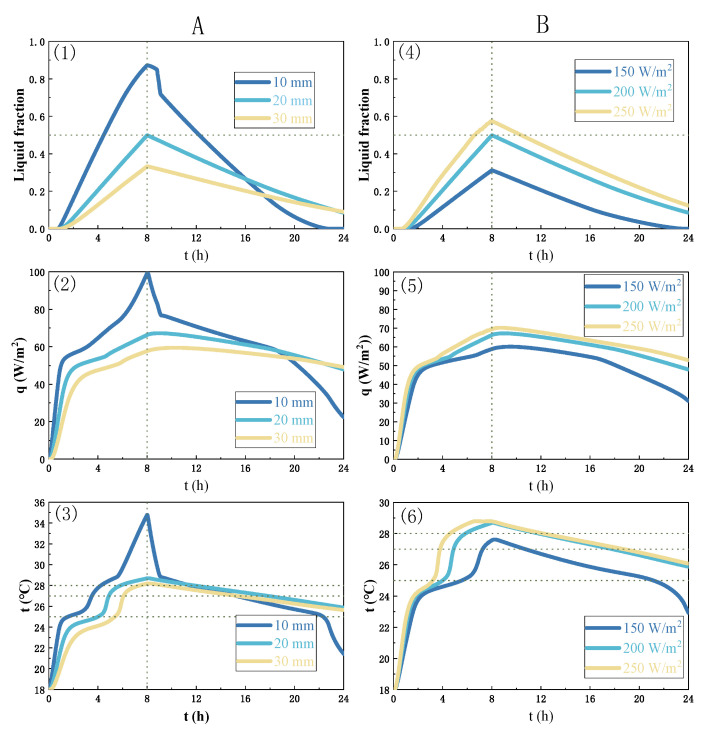

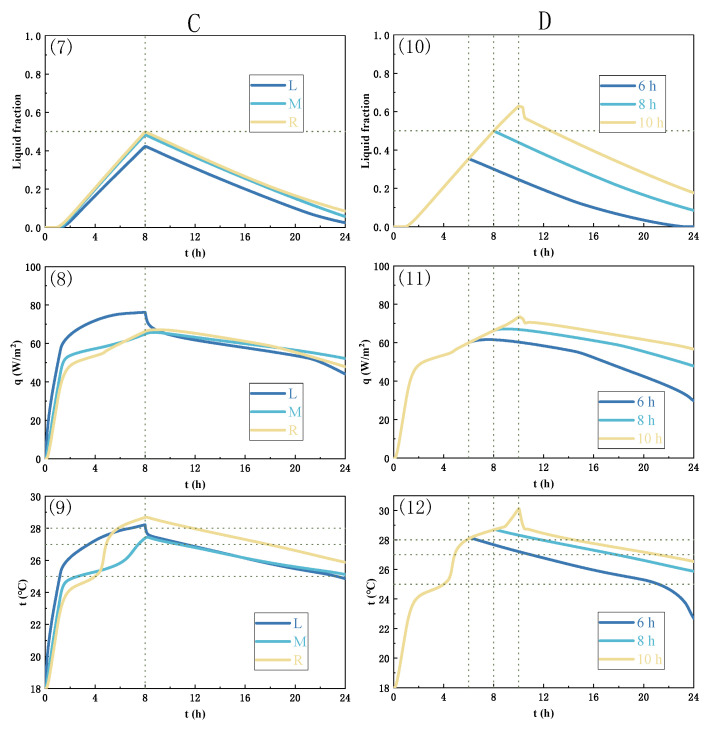
Numerical simulation results among 4 groups and 12 case scenarios: Group A, PCM layer thickness; Group B, heating power capacity; Group C, PCM layer location with respect to the heating film; Group D, time ratio of the PCM charge-to-discharge process.

Figure 10 shows the temperature variation curves in one complete charge–discharge cycle for PCM wall modules with different impact design parameters among the aforementioned 4 groups and 12 case scenarios: (1) Group A, PCM layer thickness; (2) Group B, heating power capacity; (3) Group C, PCM layer location with respect to the heating film; (4) time ratio of the PCM charge-to-discharge process.

First, a thin PCM layer can decrease the time required for the surface temperature of the decorative layer to reach a comfortable temperature. For a 10 mm PCM, the average temperature of the surface of the finish layer can be as high as 34.8 °C, which exceeds the 32 °C threshold. Therefore, if the thickness of the PCM is 10 mm, the heating power or the heating time must be reduced to lower the surface temperature. Similarly, if the PCM is 30 mm thick, then the heating power or heating time must be increased.

Secondly, the PCM melting trend is similar for different heating powers in terms of increased heating capacity leading to a faster melting speed. For the studied case, the PCM melting speed reaches the maximal value when the heating power equals 250 W/m^2^, with a maximal liquid fraction of 0.57 at 8 h during a 24 h cycling periodicity. The heating power is approximately 1.67 and 1.25 times higher, and the maximal liquid fraction is 1.84 and 1.15 times higher than the other two cases, respectively. A heating power of 250 W/m^2^ releases the largest amount of stored heat in 24 h.

Thirdly, according to Equation (11), the overall heating efficiency can be calculated and compared, with efficiencies of 71.4%, 84.8%, and 98.6% for heating powers of 250 W/m^2^, 200 W/m^2^, and 150 W/m^2^, respectively. Therefore, during a 24 h cycle, increased heating power may contribute a reduction in overall heating efficiency. However, 150 W/m^2^ is not necessarily an optimal choice, as the heating of the next day starts at the end of the previous day’s cycle, and 200 W/m^2^ and 250 W/m^2^ are preferrable starting temperatures for the next day. The heat release curves of starting temperatures of 200 W/m^2^ and 250 W/m^2^ are similar, although 200 W/m^2^ is associated with lower energy consumption, so it follows that in the current situation, 200 W/m^2^ heating power is preferred over 250 W/m^2^. Furthermore, the power is 150 W/m^2^ at 12.1 h, with a temperature range of 25–27 °C, which is 1.71 and 1.21 times longer than the other two cases, respectively, despite being associated with the longest preheating time.

Fourthly, the dynamic phase transition traction features show some disparities between charge and discharge processes. In the charge process (melting), the liquid fraction reaches 0.35 at 6 h, 0.5 at 8 h, and 0.63 at 10 h. In the discharge process (solidification), the liquid fractions in the 6 h case and the 8 h case exhibit the same trend of a slow decrease, whereas the liquid fraction of the PCM at 10 h exhibits a sharp decrease followed by a slow decrease. The maximum values of heat flow for the three cases are 61.6 W/m^2^, 67.2 W/m^2^ and 73.5 W/m^2^, with heating efficiencies of 99.5%, 84.8%, and 72.1%, respectively. The maximal values of the average surface temperature of the proposed PCM wall for the three cases are assessed to be 28.1 °C, 28.7 °C, and 30.1 °C, respectively, which are all acceptable for indoor thermal comfort requirements. The lengths of time in the temperature range of 25 °C to 27 °C are 11.1 h, 7.1 h, and 3.7 h for the three cases, respectively. Therefore, 6 h of heating is preferrable to a 24 h charge–discharge cycle.

**Figure 11:**
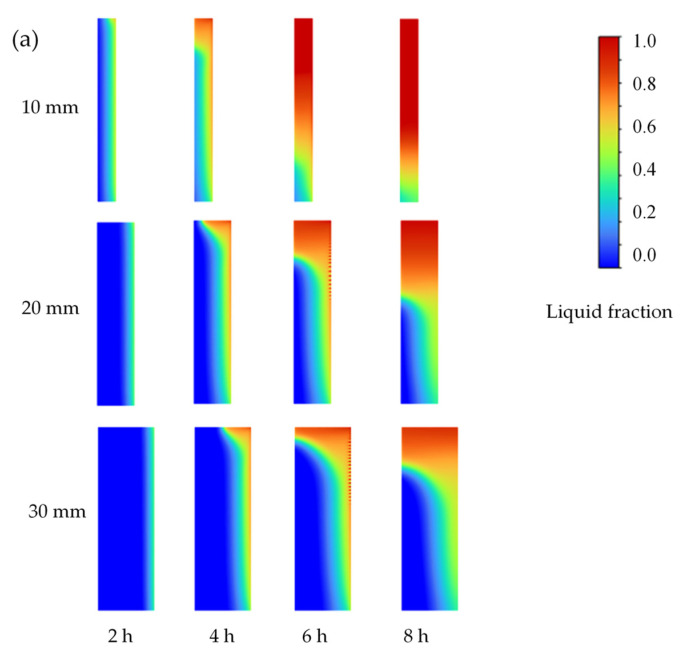

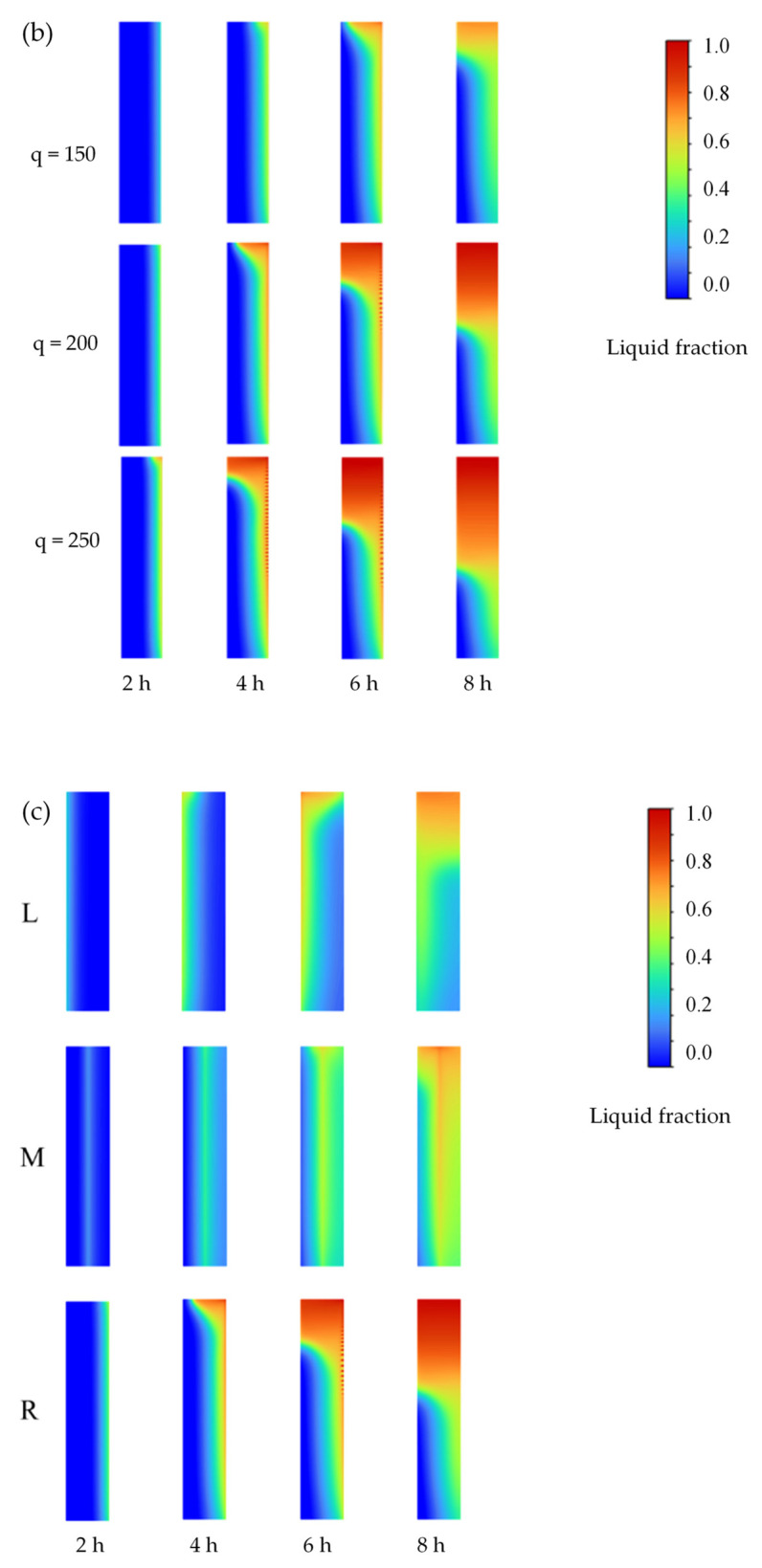
Contours of liquid fraction variations during phase transition processes with different (**a**) PCM layer thicknesses, (**b**) heating powers, and (**c**) PCM layer locations with respect to the heating film (L, left; M, middle; R, right).

In order to further illustrate the dynamic phase transition processes with consideration of the non-linearity and non-uniformity of solid and liquid PCM composites, Figure 11 shows the results of ANASYS simulation according to the established heat transfer models, depicting the contours of a liquid fraction of PCM with different deign parameters (PCM layer thickness, heating power, and location with respect to the heating film).

The solid–liquid interface shows a tilting trend due to the buoyancy force, and the tilting direction is opposite to the melting process due to the density changes for the solid–liquid PCM composite. With respect to the influence to the PCM layer thickness, 10 mm of PCM melts the fastest, and the tilting line of the solid–liquid interface basically disappears at 8 h, and approximately three-quarters of it melts completely. When the heating film layer is located on the left side of the PCM, most of the heat of the electric heating film is directly transferred to the room through the decorative layer, so the PCM absorbs less heat and melts slowly, with a low melting rate. When the heating film is located in the middle, in the first 6 h, the PCM melts evenly on the left and right sides and is basically symmetrically distributed. After 6 h, the melting rate of the PCM on the right side of the heating file is slightly higher than that on the left side because the heat on the left side is transmitted to the room through the decorative layer. In contrast, the right side comprises insulation material, which is an adiabatic boundary condition, so more heat is absorbed by the PCM on the right side of the heating layer. When the heating layer is located on the right side of the PCM, the melting speed of the PCM is the fastest.

The melting rate and the liquid fraction of the PCM are highest when the heating film is located on the right side, but the difference is not significant relative to a centered heating film layer. The PCM melting is more uniform when the heating film layer is centered, and the heat storage capacity of the PCM is more fully utilized. Furthermore, during the heat storage process, the heating film located on the left side discharges the most heat, followed by the heating film located in the middle and the heating film located on the right side; however, during the heat release process, the difference in heat exchange between the three cases is not significant, and the order is completely opposite. In general, under the same heating power, the heating film on the left side discharges the most heat in 24 h, and its heating efficiency is the highest at 90.5%, whereas similar heating efficiencies of 86.0% and 84.8% occur when the electric heating is located on the left side and in the middle, respectively; however, the heat flow density curve of the heating film located in the middle is smoother and the exothermic heat is more uniform. In addition, when the PCM is located on the left side, the surface temperature of the decorative layer increases most rapidly in the preheating stage. When the film is located in the middle, no overheating occurs, and overheating is most serious when the film is located on the right side. When the heating film is located in the middle, the surface temperature of the PCM wall ranges between 25 and 27 °C for the longest time, i.e., 18.2 h, which is 1.26 times longer than the left-side case and 2.57 times longer than the right-side case.

## 4. Conclusions and Prospects

In this paper, sodium–urea composites were investigated as PCM components, and a novel molecular PCM wall panel module (PCM wall) was proposed for building thermal energy storage. Then, based on the enthalpy–porosity method, a mathematical heat transfer model of the proposed PCM wall was established, and numerical simulation studies on the dynamic charge and discharge characteristics of the module were conducted using ANSYS software. The influencing factors and optimization solutions for the heat transfer characteristics of a PCM wall were analyzed, providing a theoretical basis for the design and application of such systems and equipment modules. Through experimentation and numerical case analysis of heat transfer in a PCM wall, the following main conclusions were drawn.

(1) An increased urea fraction leads to a reduction in melting temperature. For 30% urea composite, the melting temperature ranges from 28 to 30 °C, approaching the indoor thermal comfort level, with enhanced thermal stability during cycles;
(2) With decreasing PCM layer thickness, the melting time is reduced, and released heat capacity increases. However, reducing the PCM layer thickness may also increase overheating risk, leading to considerable fluctuation of the heat flow and surface temperature;
(3) Increased heating power contributes to increased PCM melting speed. For the studied case, a 20 mm thick PCM layer with 150 W/m^2^ heating power can maintain the surface temperature within the comfort range for approximately half the time in a charge–discharge cycle;
(4) Placing the heating film in the middle of the PCM wall unit can improve the overall heat efficiency and PCM melting uniformity, shortening the time required to reach the thermal comfort temperature range.

The present work focuses on thermal performance analysis of the proposed composite PCM wall through heat transfer modeling, with emphasis on investigation of dynamic phase transition features during charge–discharge cycles. In practical PCM applications, the thermal responses are influenced by various factors, such as building load demands, system configurations, cycling periodicity, etc. Such limitations should be addressed in future studies in the next research stage, including but not limited to (1) prototype experimentation and testing in real buildings and comparison with other available PCMs or corresponding application types; (2) investigation of the influences and improvement of the thermophysical, mechanical, and thermal–economic properties of the proposed PCM wall; and (3) generality and feasibility analysis of the established dynamic heat transfer models for other materials and field conditions. Although the specific results obtained in the case studies presented herein may not correspond to all application conditions, this work can provide theoretical and methodological support for thermal performance estimation of PCM–TES systems and offer initial guidance for PCM optimization design in building applications.

## Figures and Tables

**Table 1:** Solid–liquid PCMs used in building sectors [13,14,17].

	PCM	Melting Temperature *T*_m_ (°C)	Enthalpy *H*_m_ (kJ/kg)
Organic	Paraffin	25–30	150
	Butyl stearate CH_3_(CH_2_)_16_COO(CH_2_)_3_CH_3_	18–23	140
	N-octadecane CH_3_(CH_2_)_16_CH_3_	22.5–26.2	205
	Dodecanol CH_3_(CH_2_)_11_OH	17.5–23.3	188
Inorganic	Potassium fluoride KF·4H_2_O	18.5–19	231
	Calcium chloride CaCl_2_·	29.7	171
	Sodium sulphite Na_2_S_2_O_3_·	40	210
	Sodium acetate CH_3_COONa	45–55	240

**Table 2:** Structure and main thermophysical properties of the proposed modular PCM wall unit [3].

Layer	Decoration	PCM	Frame	Insulation
Material	Wood fiber	Sodium acetate–urea	Nanomontmorillonite fiber composites	Extruded polystyrene
Thickness (mm)	8	20	3	20
Solid/liquid density, *ρ* (kg/m^3^)	1000	1460/1480	2000	35
Thermal solid/liquid conductivity, *k* (W/m·K)	0.34	1.2/0.56	2002.5	1380
Specific solid/liquid heat capacity, *c_p_* (J/kg·K)	2510	2410/2720	0.45	0.03
Thermal expansion coefficient, *β* (1/K)		0.00044		
Dynamic viscosity, *μ* (Pa·s)		0.00324		
Melting point, *T_m_* (K)		301.15–305.15		
Latent heat, *h_sf_* (kJ/kg)		200		

**Table 3:** Case scenarios of a modular PCM wall unit.

Group	Case	d (mm)	q (W/m^2^)	Position	*t*_charge_/*t*_discharge_
A	1	10	200	R	8/16
	2	20	200	R	8/16
	3	30	200	R	8/16
B	4	20	150	R	8/16
	5	20	200	R	8/16
	6	20	250	R	8/16
C	7	20	200	L	8/16
	8	20	200	M	8/16
	9	20	200	R	8/16
D	10	20	200	L	6/18
	11	20	200	M	8/16
	12	20	200	R	10/14

## Data Availability

The data presented in this study are available upon request from the corresponding author.
